# 克唑替尼治疗ALK阳性晚期非小细胞肺癌患者的疗效观察

**DOI:** 10.3779/j.issn.1009-3419.2015.10.03

**Published:** 2015-10-20

**Authors:** 静 赵, 坤 张, 力予 张, 红 王

**Affiliations:** 100071 北京，中国人民解放军军事医学科学院附属医院肺部肿瘤科 Department of Lung Oncology, Affiliated Hospital of the PLA Military Academy of Medical Sciences, Beijing 100071, China

**Keywords:** 肺肿瘤, 克唑替尼, 多西他赛, ALK, Lung neoplasms, Crizotinib, Docetaxel, ALK

## Abstract

**背景与目的:**

间变淋巴瘤激酶(anaplastic lymphoma kinase, *ALK*)融合基因的发现促进了非小细胞肺癌(non-small cell lung cancer, NSCLC)分子靶向药物的发展, 是继表皮生长因子受体之后NSCLC中重要的治疗靶点。本研究将探索克唑替尼治疗ALK阳性中晚期NSCLC患者的临床疗效。

**方法:**

将28例ALK阳性中晚期NSCLC患者随机分为克唑替尼组(*n*=14)和化疗组(*n*=14), 克唑替尼组给予克唑替尼胶囊250 mg/粒, 一次1粒, 每日2次; 化疗组给予多西他赛75 mg/m^2^静脉滴注1 h, 每3周1次, 3周为1个疗程, 至少用药3个疗程, 随访12个月, 观察两组的临床疗效。

**结果:**

克唑替尼组患者有效率为64.29%, 明显高于化疗组的21.43%(*P*=0.026);克唑替尼组稳定率为85.71%, 明显高于化疗组的40.86%(χ^2^=5.600, *P*=0.018);克唑替尼组患者中位无进展生存时间(progression free survival, PFS)为7.0个月, 较化疗组患者中位PFS为4.0个月长(*P*=0.002)。

**结论:**

克唑替尼在ALK阳性中晚期NSCLC患者的临床疗效优于常规化疗, 可延长中位PFS, 提高患者生存质量。

## Abstract (en)

**Background and objective:**

The aim of this study is to explore clinical efficacy of crizotinib in advanced anaplastic lymphoma kinase (ALK) positive non-small cell lung cancer.

**Methods:**

Twenty-eight patients with advanced non-small cell lung cancer habouring ALK positive were randomly divided into crizotinib group (*n*=14) and chemotherapy group (*n*=14).Patients in the crizotinib group were receive oral treatment with crizotinib (250 mg) twice daily.Patients in the chemotherapy group were administrated docetaxel injection (75 mg/m^2^) every three weeks and every patient was treated at least 3 period.Then clinical efficacy was observed after 12 mo followed-up.

**Results:**

Effective rate of patients in the crizotinib group was 64.29%.It was significantly higher than that of the chemotherapy group (21.43%)(*P*=0.026).The stable rate of patients in the crizotinib group was 85.71%.It was significantly higher than that of the chemotherapy group 40.86% (χ^2^=5.600, *P*=0.018).Median progression free survival (PFS) of the crizotinib group was 7.0 mo.It was longer than that of the chemotherapy group (4.0 mo)(*P*=0.002).

**Conclusion:**

Crizotinib is superior to standard chemotherapy in patients with previously treated, advanced ALK positive non-small cell lung cancer.The median PFS of patients is shorter.It can improve the quality of life about patients.

肺癌是源于支气管粘膜或腺体的恶性肿瘤, 其发病率位于肿瘤的第一位, 由于早期诊断不足, 预后较差^[1]^。按病理学分类可分为:非小细胞肺癌(non-small cell lung cancer, NSCLC)和小细胞肺癌(small cell lung cancer, SCLC)两类, 其中NSCLC占肺癌的75%-80%, 5年生存率仅为10%-15%左右^[2]^。目前晚期NSCLC的治疗方法主要为化疗, 但NSCLC患者化疗的效果已进入平台期^[3]^, 靶向药物治疗正处于研究的早期阶段。有研究表明棘皮动物微管相关蛋白样4(echinoderm microtubule-associated protein-like 4, *EML4*)-间变淋巴瘤激酶(anaplastic lymphoma kinase, *ALK*)为NSCLC的驱动基因^[4]^, 而克唑替尼是EML4-ALK的选择性抑制剂^[5]^。自2011年美国食品药品管理局批准克唑替尼治疗*ALK*基因表达异常的NSCLC以来^[6]^, 国内外学者们对其疗效及副反应进行了观察, 国内研究起步相对较晚, 需要对其临床效果作进一步评价, 同时探讨其对ALK阳性晚期NSCLC患者的生存状况的影响。本研究对克唑替尼治疗ALK阳性晚期NSCLC患者的临床疗效、生存质量、无进展生存时间(progression-free survival, PFS)及不良反应进行观察, 现报道如下。

## 1 资料与方法

### 1.1 一般资料

选取2012年1月-2013年12月于中国人民解放军军事医学科学院附属医院就诊且诊断为NSCLC中晚期患者28例, 纳入标准:①经组织学或细胞学检查诊断确诊为NSCLC, 经一线或二线常规化疗进展后, *ALK*基因荧光原位杂交技术(fluorescence *in situ* hybridization, FISH)检测为阳性; ②体力状况:卡氏体能状态(Karnofsky performance status, KPS)评分≥70分; ③年龄18岁以上; ④经计算机断层扫描(computed tomography, CT)或磁共振成像(magnetic resonance imaging, MRI)扫描有可测量病灶; ⑤预计生存期大于3个月; ⑥心、肝、肾及骨髓造血功能无异常。排除标准:①合并其他肿瘤的患者; ②合并糖尿病、高血压及心脏病等; ③妊娠或哺乳期妇女; ④不宜参加本研究的其他情况。所有参与患者均签署知情同意书。将患者按照就诊先后随机分为克唑替尼组和化疗组, 每组各14例, 两组患者的一般情况见表 1, 经比较差异无统计学意义, 有可比性。

**1:** 两组患者一般情况比较 Comparison of general condition of patients in two groups

Characteristics		Crizotinib group (*n*=14)	Chemotherapy group (*n*=14)	*t*/χ^2^/*Z*	*P*
Age (yr)		55.33±12.68	58.09±13.15	0.565	0.577
Gender	Male	8	9	0.15	0.699
	Female	6	5		
Pathological type	Squamous	8	10	0.622	0.430
	Adenocarcinoma	6	4		
Stage	Ⅲa	4	5	0.389	0.734
	Ⅲb	6	6		
	Ⅳ	4	3		
Karnofsky performance status		81.83±5.29	84.01±6.12	1.008	0.323

### 1.2 治疗方法

克唑替尼组患者给予克唑替尼胶囊250 mg/粒, 一次1粒, 每日2次; 化疗组给予多西他赛75 mg/m^2^静脉滴注1 h, 每3周1次, 化疗前一天给予地塞米松口服预防过敏反应, 3周为1个疗程。两组至少治疗3个疗程, 治疗期间定期检查患者肝肾功能、血常规、心电图及CT, 观察评价不良反应, 随访12个月。

### 1.3 观察指标

疗效评价标准:依据实体瘤疗效评价标准(Response Evaluation Criteria in Solid Tumors, RECIST)1.0标准^[7]^, 分为完全缓解(complete response, CR), 部分缓解(partial response, PR), 稳定(stable disease, SD), 进展(progressive disease, PD), 有效率=(CR+PR)例数/总例数。生存质量:按照世界卫生组织(World Health Organization, WHO)卡氏评分标准:KPS评分较治疗前增加10分为提高, KPS评分较治疗前得分改变10分以内为稳定, KPS评分较治疗前减少10分为下降, 稳定率=(稳定+提高)例数/总例数。PFS为治疗开始至疾病进展或尚未进展的末次随访的时间(月)。

### 1.4 统计学方法

采用SPSS 17.0软件进行分析, 计量资料采用*t*检验, 计数资料用χ^2^检验, 生存曲线绘制采用*Kaplan-Meier*法, 以*P* < 0.05为差异有统计学意义。

## 2 结果

### 2.1 临床疗效比较

克唑替尼组CR占28.57%(4/14), PR占35.71%(5/14), SD占28.57%(4/14), PD占7.14%(1/14), 有效率为64.29%;化疗组CR占0, PR占21.43%(3/14), SD占35.71%(5/14), PD占42.86%(6/14), 有效率为21.43%;克唑替尼组疗效明显高于化疗组, 两组差异有统计学意义(*P*=0.026)。

### 2.2 两组患者的KPS生存质量比较

克唑替尼治疗后患者的生存质量提高者3例, 稳定者9例, 下降者2例, 克唑替尼组稳定率为85.71%, 化疗后生存质量提高者1例, 稳定者5例, 下降者8例, 化疗组稳定率为40.86%, 经卡方检验差异有统计学意义(χ^2^=5.600, *P*=0.018)。

### 2.3 两组患者PFS生存情况

对两组患者进行12个月的随访, 无失访患者, 克唑替尼组患者中位PFS为7.0个月, 化疗组患者中位PFS为4.0个月; 两组疗法*Log-rank*检验比较, 克唑替尼组的PFS明显长于化疗组, 差异有统计学意义(χ^2^=9.574, *P*=0.002), 见图 1。

**1:**
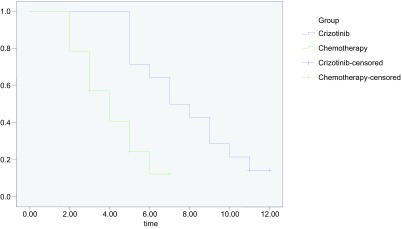
克唑替尼组与化疗组患者PFS比较 Comparison of progression free survival (PFS) of patients in two groups

### 2.4 不良反应情况

克唑替尼组不良反应主要有视觉效应、恶心、便秘及转氨酶升高, 无骨髓抑制现象, 不良反应发生率为42.86%(6/14);化疗组主要不良反应有脱发、恶心、呕吐、腹泻及转氨酶升高, 不良反应发生率为52.14%(8/14);两组患者不良反应发生率经卡方检验比较差异无统计学意义(χ^2^=0.571, *P*=0.450), 所有患者均能耐受并坚持治疗结束, 具体见表 2。

**2:** 克唑替尼组与化疗组患者不良反应比较 Comparison of adverse event of patients from both groups

Adverse event	Crizotinib group (*n*=14)	Chemotherapy group (*n*=14)
Visual disorder	2	0
Nausea	1	2
Vomit	0	1
Constipation	1	0
Diarrhea	0	1
Drug-induced liver injury	2	1
Alopecia	0	3

## 3 讨论

自从Soda^[4]^在NSCLC患者的标本中发现*EML4-ALK*融合基因以来, 针对*ALK*基因阳性的NSCLC患者靶向药物治疗越来越受到人们的关注, 目前使用的药物主要为克唑替尼。克唑替尼(分子式:C21H22CL2FN5O)是口服的ALK竞争性抑制剂^[5]^, 主要通过剂量依赖的方式抑制细胞内ALK与c-Met激酶磷酸化, 从而抑制肿瘤细胞的增殖作用, 促进肿瘤细胞的凋亡^[7]^。克唑替尼Ⅰ期临床试验^[8]^结果表明, 可以明显提高ALK阳性患者的中位生存时间和生存率。

本研究对ALK阳性中晚期NSCLC患者分别给予克唑替尼(靶向治疗)和多西他赛(化疗药物)治疗, 结果表明:克唑替尼治疗患者的有效率为64.29%, 明显高于多西他赛治疗组, 克唑替尼组患者中位PFS也明显长于化疗组(多西他赛), 与国内学者们的研究^[9, 10]^相一致, 这可能和克唑替尼选择性抑制ALK, 同时与克唑替尼上调Bim基因的表达水平和下调抗凋亡蛋白Bcl-2、Bcl-xL有关, 最终诱导细胞凋亡^[11]^。本研究同时观察两组患者的生存质量, 发现克唑替尼组患者的生存质量(KPS评分)明显高于化疗组, 说明克唑替尼能明显改善患者的生存质量, 对患者的继续生存及治疗具有积极的影响作用。所有患者的不良反应普遍较轻, 均坚持完成治疗, 克唑替尼主要的不良反应为视觉效应、恶心、便秘及转氨酶升高, 而多西他赛的主要不良反应为脱发、恶心、呕吐、腹泻及转氨酶升高, 两组的不良反应发生情况无明显差异。国外Ⅱ期、Ⅲ期临床试验结果^[12, 13]^表明, 克唑替尼对ALK阳性晚期NSCLC疗效明显优于化疗, 由此可见克唑替尼治疗ALK阳性晚期NSCLC患者临床疗效可靠, 值得在临床推广应用。

有研究^[14]^表明克唑替尼可以使*EML4-ALK*基因中C1156Y和L1196M二次突变, 使克唑替尼对于原有靶点的亲和力减弱, 从而产生耐药性。本研究尚未对克唑替尼的耐药性进行观察, 以明确耐药的机制, 这是本次研究的不足之处。本研究中克唑替尼组部分进展患者入组了一项“既往接受克唑替尼治疗的*ALK*重排(ALK阳性)晚期NSCLC)中国成年患者口服LDK378的一项多中心、开放性、单臂Ⅰ期/Ⅱ期研究”, 以评价LDK378在克唑替尼耐药ALK阳性晚期NSCLC患者中的疗效, 目前此项研究正在进行中。国外LDK378 Ⅰ期临床研究^[15]^报道显示, 130例具有ALK重排的晚期NSCLC患者, 其中114例服用LDK378至少400 mg/d的患者, 有效率为58%, PFS为7个月, 83/122例(68%)之前接受过克唑替尼治疗的患者客观缓解率为56%。

国外研究^[16]^表明, ALK阳性晚期NSCLC患者中, 克唑替尼明显优于传统含铂两药治疗, 目前国内克唑替尼一线用于治疗ALK阳性晚期NSCLC的报道还很少, 二线治疗中的明显优势将促进克唑替尼在一线治疗中的临床研究。郑明英等^[17]^报道12例患者克唑替尼治疗4周客观缓解率达91.67%, 明显高于既往报道。

综上所述, 克唑替尼治疗ALK阳性晚期NSCLC患者临床效果好, 患者PFS长, 生存质量得分高, 不良反应不明显, 是治疗ALK阳性晚期NSCLC患者的有效方法。
